# Complete Genome Sequence of Klebsiella pneumoniae Myophage Magnus

**DOI:** 10.1128/MRA.01049-19

**Published:** 2019-09-26

**Authors:** Laura E. Acevedo Ugarriza, Jordyn Michalik-Provasek, Heather Newkirk, Mei Liu, Jason J. Gill, Jolene Ramsey

**Affiliations:** a Center for Phage Technology, Texas A&M University, College Station, Texas, USA; b Department of Animal Science, Texas A&M University, College Station, Texas, USA; Queens College

## Abstract

Bacteriophage Magnus infects Klebsiella pneumoniae, a Gram-negative pathogen whose multidrug-resistant strains are a public health issue. Here, we describe the annotation of the 157,741-bp Magnus genome and its similarity to other myophages.

## ANNOUNCEMENT

Klebsiella pneumoniae is an opportunistic, Gram-negative enteric bacterium. Carbapenemase-producing K. pneumoniae (KPC) strains are resistant to carbapenem antibiotics and are an emerging cause of nosocomial and systemic human infections (1). Phage therapy offers a potential treatment for these multidrug-resistant infections. In this report, we describe the genome of bacteriophage Magnus.

Bacteriophage Magnus was isolated from filtered samples (filter size, 0.2 μm) collected at a wastewater treatment facility in Austin, TX, against K. pneumoniae strain 39427 (BioSample number SAMN06218024) (2). The host was grown aerobically in tryptic soy broth or agar (Difco) at 37°C, and phage propagation was done using the soft agar overlay method (3). Phage genomic DNA was purified using the Promega Wizard DNA clean-up system, according to Summer’s modification to the shotgun library preparation protocol (4). A paired-end 250-bp library for sequencing was prepared with a TruSeq Nano low-throughput kit and then sequenced by an Illumina MiSeq platform using v2 500-cycle chemistry. The quality of 373,956 total Illumina reads in the index was examined using FastQC (www.bioinformatics.babraham.ac.uk/projects/fastqc). Quality-controlled reads were trimmed using the FASTX-Toolkit v0.0.14 (http://hannonlab.cshl.edu/fastx_toolkit/) and then assembled into a single contig of circular assembly at 146-fold coverage using SPAdes v3.5.0 (5). The contig was confirmed to be complete by PCR (forward primer, 5′-ATGTATTAGTCGCGCCGTATG-3′; reverse primer, 5′-TGGTGCCAGCGTCAATAA-3′), with products from the PCR amplification sequenced by Sanger sequencing. Genes were called using ARAGORN v2.36, Glimmer v3.0, and MetaGeneAnnotator v1.0 (6–8). Terminators (Rho independent) were predicted with the TransTermHP tool v2.09 (9). Gene functions were predicted using InterProScan v5.22-61 (10) and BLAST v2.2.31 versus NCBI nonredundant, UniProtKB Swiss-Prot, and UniProtKB TrEMBL databases, with a 0.001 maximum expectation cutoff (11, 12). Bioinformatic analyses and sequence annotation were performed using tools available in the Center for Phage Technology Galaxy and Web Apollo instances (https://cpt.tamu.edu/galaxy-pub/) (13, 14). Unless otherwise stated, all tools were executed using default parameters. For transmission electron microscopy, Magnus was negatively stained with 2% (wt/vol) uranyl acetate, and then the morphology was observed at the Texas A&M Microscopy and Imaging Center (15).

Magnus is a myophage that has a 157,741-bp genome and a coding density of 92%, with 212 predicted protein-coding genes, 71 of which have a predicted function. The Magnus genome also contains 5 tRNA genes (Arg, Met, Gln, Asn, and Ser). Its G+C content was determined to be 46.3%. It was predicted by PhageTerm that Magnus uses a headful packaging system (16).

By BLASTp and progressiveMauve analyses, Magnus has 75.15% nucleotide identity to, and shares 146 similar proteins with, *Klebsiella* phage 0507KN21 (GenBank accession number AB797215) (17). Magnus also shares 172 proteins with *Serratia* phage vB_Sru_IME250 (GenBank accession number KY073123) and *Shigella* phage Ag3 (GenBank accession number FJ373894), all classified within the Ackermannviridae. Additionally, Magnus contains at least 27 genes with significant amino acid similarity to type phages T4 (GenBank accession number NC_000866) and K (GenBank accession number KF766114), including core structural and replication proteins. The large terminase gene (NCBI accession number QEG07939) harbors an intein element.

### Data availability.

The genome sequence and associated data for phage Magnus were deposited under GenBank accession number MN045230, BioProject accession number PRJNA222858, SRA accession number SRR8869229, and BioSample accession number SAMN11360383.
